# Valorization of Cheese and Tofu Whey through Enzymatic Synthesis of Lactosucrose

**DOI:** 10.1371/journal.pone.0139035

**Published:** 2015-09-25

**Authors:** Marta Corzo-Martinez, Alice Luscher, Blanca de las Rivas, Rosario Muñoz, F. Javier Moreno

**Affiliations:** 1 Instituto de Investigación en Ciencias de la Alimentación, CIAL (CSIC-UAM), CEI (UAM+CSIC), C/ Nicolás Cabrera 9, 28049, Madrid, Spain; 2 Instituto de Ciencia y Tecnología de Alimentos y Nutrición, ICTAN (CSIC), C/ Juan de la Cierva 3, 28006 Madrid, Spain; University of Insubria, ITALY

## Abstract

This work deals with the development of a new bioprocess for the efficient synthesis of lactosucrose, a potential prebiotic oligosaccharide with a high value-added, from two important and inexpensive agro-industrial by-products such as tofu whey and cheese whey permeate. The bioconversion is driven by the ability of the enzyme levansucrase SacB from *Bacillus subtilis* CECT 39 to transfructosylate lactose contained in the cheese whey permeate by using not only sucrose but also raffinose and stachyose, which are present in considerable amounts in the tofu whey, as suitable donors of fructosyl moieties. The maximum lactosucrose concentration obtained from both by-products was 80.1 g L^-1^ after a short reaction time 120 min at 37°C, leading to productivity and specific productivity values of 40.1 g lactosucrose L^-1^ h^-1^ and 80.1 mg lactosucrose U enzyme^−1^ h^−1^, respectively. Findings contained in this work could provide a new strategy to valorize agro-industrial by-products as cheese whey permeate and, specially, tofu whey by means of their use as renewable resources in the enzymatic synthesis of bioactive oligosaccharides.

## Introduction

Over the recent years, the demand in the international market for new food ingredients providing not only the basic nutritional value but also benefits that can contribute to improve consumer well-being is increasing. That has promoted the industrial development and commercialization of a new generation of foods called functional foods. Among them, a great attention has been paid to prebiotic oligosaccharides, which are defined as “nondigestible oligosaccharides that beneficially affect the host by selectively stimulating the growth and/or activity of one or a limited number of bacteria in the colon” [1]. The importance of the colonic microbiota in human health and nutrition is well-known. The human large intestine plays an important role as a nutritional organ mainly due to the metabolic activities of the resident microbiota. In addition, a large number of gut disorders, such as constipation, diarrhea, and colorectal cancer [2, 3], are strongly related to the composition of colonic microbiota [4]. For this reason, prebiotics are a rapidly growing ingredient category for formulating functional dietary supplements, foods and beverages.

Nowadays, the main oligosaccharides commercialized as prebiotics are inulin, fructooligosaccharides (FOS) and galactooligosaccharides (GOS) [5]; however, a notable number of carbohydrates such as isomaltooligosaccharides [6], soya oligosaccharides [7], xylooligosaccharides [8] and lactosucrose [9, 10] have demonstrated to potentially possess prebiotic effect. The latter, lactosucrose (O-β-D-galactopyranosyl-(1–4)-O-α-D-glucopyranosyl-(1–2)-β-D-fructofuranoside), also known as 4^G^-β-D-galactosylsucrose or lactosylfructoside, is a functional food ingredient with important industrial applications due to its physical-chemical properties and physiological functions. Concretely, lactosucrose is a non-digestible carbohydrate [11] and, therefore, has a great potential as sweetener suitable for low calorie foods. In addition, it has been reported its ability to i) promote the intestinal mineral absorption; ii) protect the intestinal conditions, underlining a potential treatment effect on inflammatory bowel disease, Crohn’s disease or ulcerative colitis; iii) prevent constipation; iv) inhibit body fat accumulation and prevent obesity [12–15], among other health benefits [16]. Despite of that, nowadays, lactosucrose commercialization and use is largely confined to Japan [16], where it has been approved as an important functional food ingredient for its inclusion in more than 30 foodstuffs and beverages for specified health use (FOSHU).

Lactosucrose scarcely exists in nature, being enzymatically synthetized from two disaccharides as lactose (O-β-D-galactopyranosyl-(1–4)-D-glucopyranose) and sucrose (O-α-D-glucopyranosyl-(1–2)-β-D-fructofuranoside). These disaccharides are the major constituents of two important by-products of the food industry; cheese whey permeate and tofu whey, derived from the cheese and tofu manufacturing, respectively. Disposal of such by-products to waste waters is highly polluting, representing, thus, their friendly environmental management a high cost for the food industry. Consequently, in recent years, there is an increasing interest by searching new alternatives to expand the use of by-products in an efficient way. In this sense, cheese whey permeate has been used as source of lactose for synthesis of GOS by enzymatic transgalactosylation [17, 18]. Likewise, different ways of production of new prebiotic oligosaccharides, involving simple or combined processes of enzymatic transglycosylation and chemical isomerization, have been recently developed [19, 20]. However, tofu whey has been traditionally considered as a low-value residue [21, 22] and, as far as we know, the main valorization strategy existing until now for tofu whey management consists in its use as substrate for the production of “starters” to ferment soya products [21, 23].

This work describes a new bioprocess for the efficient conversion of two abundant and inexpensive agro-industrial by-products, such as tofu whey and cheese whey permeate, into lactosucrose by a transfructosylation reaction catalysed by the enzyme levansucrase SacB from *Bacillus subtilis* CECT 39. This novel procedure to upgrade two agrofood by-products through the production of a functional value-added ingredient as lactosucrose could result in economic and environmental benefits for the agro-food industry.

## Materials and Methods

### Chemical and reagents

Reagents used for chromatographic analysis, including pure standards of fructose (Fru), glucose (Glu), sucrose (Suc), lactose (Lac), melibiose (Mel), raffinose (Raf) and stachyose (Sta), were obtained from Sigma (St Louis, MO, USA). Lactosucrose, also termed lactosyl fructoside, (LacSuc) was purchased from Wako Pure Chemical Industries (Osaka, Japan). Acetonitrile of high-performance liquid chromatography (HPLC) grade was purchased from Lab-Scan (Gliwice, Poland). All other chemicals were of analytical grade. Ultrapure water (18.2 MΩ cm, with levels of 1–5 ng mL^−1^ total organic carbon and <0.001 EU mL^−1^ pyrogen) produced in-house with a laboratory water purification system (Milli-Q Synthesis A10, Millipore, Billerica, MA, USA) was used throughout.

### Production, purification and activity assay of recombinant levansucrase enzyme

Levansucrase (EC 2.4.1.10) (accession YP_003867730) SacB from *Bacillus subtilis* CECT 39 (ATCC 6051) was overproduced in *Escherichia coli* and purified as previously indicated by Díez-Municio [24].

Fructosidase and fructosyltransferase activities of levansucrase were measured as a function of the amounts of glucose and fructose released from sucrose. Fructosidase activity was expressed as the amount of free glucose. The fructose transferred to levan (fructosyltransferase activity) was defined as the difference between the amount of released glucose and fructose. Enzyme activity measurements were repeated three times, and the experimental error was less than 5%. Levansucrase expressed a specific fructosidase activity of 2.9 units per milligram (U mg^-1^), where 1 unit is defined as the amount of enzyme releasing 1 μmol of glucose per minute at a working temperature of 37°C and a sucrose concentration of 100 g L^−1^ at pH 6.0 (50 mM potassium phosphate buffer). The fructosyltransferase activity was 1.9 U mg^−1^, where 1 unit is the amount of enzyme required to perform the transfer of 1 μmol of fructose per minute at a working temperature of 37°C and a sucrose concentration of 100 g L^−1^ at pH 6.0 (50 mM potassium phosphate buffer). These activity values should be taken only for guidance because the ratio between hydrolysis and transglycosylation activities of glycansucrases largely depends on the initial concentration of sucrose.

### Physical-chemical characterization of cheese whey permeate and tofu whey

Bovine cheese whey permeate (CWP) powder and fresh tofu whey (TW) were kindly supplied by the industries García Baquero (Alcázar de San Juan, Spain) and Natursoy (Barcelona, Spain), respectively. TW was freeze-dried prior to its physical-chemical characterization.

The dry matter (DM) content was gravimetrically determined by drying the samples in a conventional oven at 100°C until constant weight (~48 h), according to the AOAC method [25].

For ion composition of both wheys (CWP and TW), either a semiquantitative analysis or a quantitative analysis of the most representative elements present in samples were carried out using an ICP-MS ELAN 6000 Perkin-Elmer Sciex instrument at the Servicio Interdepartamental de Investigacioón of Universidad Autónoma of Madrid (SIdI-UAM). Estimation of element concentration was done using the external calibration method and internal standards to correct instrumental drift, following the method previously described [26].

The total protein content of both by-products was determined using the Kjeldahl method [25].

The pH of both wheys was measured using a pH meter (MP 230, Mettler-Toledo, Barcelona, Spain) at a concentration of 50 mg mL^−1^ in the case of the CWP, whereas the pH of TW was measured before freeze-drying. Finally, the determination of carbohydrate content, as well as the characterization of the carbohydrate profile was performed by LC-RID. Sample preparation and the used chromatographic method are described below.

### Synthesis of lactosucrose by enzymatic transfructosylation

Lactosucrose (LacSuc) synthesis was carried out from different reaction mixtures, which were constituted by sucrose (Suc), raffinose (Raf), stachyose (Sta) or tofu whey (TW), as donors of fructosyl groups, and lactose (Lac) or cheese whey permeate (CWP), as acceptors (Table 1). Such mixtures were incubated with levansucrase SacB from *B*. *subtilis* CECT 39 (0.5 U mL^-1^ of reaction volume) at 37°C, pH 6.0 (50 mM potassium phosphate buffer) and continuous agitation at 1,350 rpm using an orbital shaker (Eppendorf Thermomixer Confort, Hauppauge, NY, USA) (optimum conditions of enzyme were according to Dìez-Municio [24]). The enzyme to substrate ratios (IU/ g) employed were: 1: 1 for model systems; 1: 1.5 and 1: 1.2 for tofu whey-lactose combinations; 1: 1.6 and 1: 1.2 for tofu whey-cheese whey permeate mixtures.

**Table 1:** Mixtures of sucrose, raffinose, stachyose or tofu whey (as fructosyl donors) and lactose or cheese whey permeate (as acceptors) used as substrates in the enzymatic synthesis of lactosucrose.

Donors (concentration, %, w/v)	Acceptors (concentration, %, w/v)
Sucrose (25)	Lactose (25)
Raffinose (25)	Lactose (25)
Stachyose (25)	Lactose (25)
Tofu whey (51.6) ^a^	Lactose (25)
Tofu whey (51.6)	Cheese whey permeate (27.8) ^c^
Tofu whey (47.3) ^b^	Lactose (12.5)
Tofu whey (47.3)	Cheese whey permeate (13.9) ^d^

^a^Equivalent to Suc:Raf:Sta (9.7:1.7:7.5).

^b^ Equivalent to Suc:Raf:Sta (9:1.6:7).

^c^ Equivalent to 25% Lac.

^d^ Equivalent to 12.5% Lac.

Evolution of lactosucrose formation was determined by taking aliquots from the reaction mixture at suitable time intervals, including 0, 30, 60, 90, 120 and 180 min. The enzyme was inactivated by heating at 100°C for 5 min.

Moreover, control experiments of the donor substrate and enzyme in the absence of acceptor, as well as of enzyme in the absence of donor and acceptor carbohydrates were carried out with the purpose of checking the formation of products derived from the donor substrate or the enzyme incubation.

All the synthesis reactions were done in duplicate.

Finally, the following defined parameters were used to assess the enzymatic synthesis of lactosucrose:

- Yield (g lactosucrose/100 g lactose added) represents the mass of lactosucrose obtained during the synthesis per unit mass of initial lactose.
- Productivity (g lactosucrose L^-1^·h^-1^) represents the concentration of lactosucrose produced per unit of reaction time.
- Specific productivity (mg lactosucrose U enzyme^-1^·h^-1^) represents the mass of lactosucrose produced per unit of fructosyltransferase activity of levansucrase SacB from *B*. *subtilis* CECT 39 added and per unit of reaction time.

For the purposes of this work, these parameters were evaluated at the time point when the maximum lactosucrose concentration was achieved, under the different combinations of donor and acceptor concentrations displayed in **Table 1**.

### Liquid chromatographic determination of carbohydrates

Chromatographic separation and quantitation of carbohydrates present in the original sample of tofu whey and samples resulting from lactosucrose synthesis was carried out by LC-RID.

Freeze-dried TW was dissolved in 50 mM potassium phosphate buffer pH 6.0 at a final concentration similar to that used in synthesis reactions (51.6%). Then, it was kept under agitation until its complete dissolution.

Before chromatographic analysis, either sample of original TW or inactivated samples resulting from enzymatic synthesis were diluted with acetonitrile:water (50:50, v:v) until a total carbohydrate concentration of ~16 mg mL^-1^ (~30-folds), filtered (0.45 μm PVDF filters, Symta, Madrid, Spain), and kept at 4°C until their analysis by HPLC with refractive index detection (RID) as described below.

HPLC analyses were carried out using an Agilent Technologies 1260 Series HPLC system (Böblingen, Germany). The separation of carbohydrates was carried out on a Kromasil column (100-NH_2_; 250mm × 4.6 mm, 5 μm particle size) (Akzo Nobel, Brewster, NY, USA) using acetonitrile/water (70:30 v/v) as mobile phase and elution in isocratic mode at a flow rate of 1 mL min^−1^ for 55 min. The injection volume was 50 μL (~ 550–850 μg of total carbohydrates). Data acquisition and processing were performed using Agilent ChemStation software. Carbohydrates in the reaction mixtures were initially identified by comparing their retention times (t_R_) with those of pure standard sugars, including Fru, Glu, Suc, Lac, Mel, LacSuc, Raf and Sta. Quantitative analysis was performed by the external standard method, using calibration curves of each pure standard in the range 0.01–2.5 mg mL^-1^. All analyses were performed in duplicate (*n* = 4), obtaining relative standard deviation (RSD) values below 10% in all cases.

### Identification of lactosucrose by gas chromatography with mass spectrometry detection (GC-MS)

Both synthesized and commercial lactosucrose were analyzed by GC-MS on an Agilent Technologies 7890A gas chromatograph coupled to a 5975C MSD quadrupole mass detector (Agilent Technologies, Wilmington, DE, USA) in order to confirm the identification of the lactosucrose. Sugar separation was performed using helium as a carrier gas at 0.8 mL min^-1^. The trimethylsilyl oxime (TMSO) derivatives were prepared as previously described [27] and separated using a HP-5 MS fused silica capillary column (30 m × 0.25mm i.d., 0.25 μm film thickness) coated with 5% phenyl methyl silicone (J&W Scientific, Folsom, CA, USA). The oven temperature was increased from 180 to 315°C at a rate of 3°C min^-1^ and held for 20 min. The injector temperature was 280°C. Injections were made in split mode (1:40). The mass spectrometer was operated in electrospray ionisation mode at 70 eV. Mass spectra were acquired using Agilent ChemStation MSD software (Wilmington, DE, USA). Identification of trimethylsilyloxime derivatives of carbohydrates was carried out by comparison of their relative retention times and mass spectra with those of standard compounds previously derivatized.

### Statistical analysis

The comparisons of means using analysis of variance (ANOVA) were made using the statistical software Microsoft Excel of Microsoft Office 2010. The differences were considered significant when P < 0.05.

## Results and Discussion

### Physical-chemical characterization of by-products

Prior to the use of cheese whey permeate (CWP) and tofu whey (TW) as substrates in the enzymatic synthesis of lactosucrose, a physical-chemical characterization of both by-products was carried out, including determination of dry matter (DM) content, total protein content, quantitative analysis of mineral salts, measurement of pH and characterization and quantification of the carbohydrate profile (Table 2).

**Table 2:** Physical-chemical characterization of industrial cheese whey permeate (CWP) and tofu whey (TW).

	CWP^a^	TW
Dry matter (DM)	94.2 ± 0.0	89.7 ± 0.8
Sucrose (% w/w DM)	^b^n.d.	21
Raffinose (% w/w DM)	n.d.	3.7
Stachyose (% w/w DM)	n.d.	16.3
Lactose (% w/w DM)	89.9	n.d.
Fructose (% w/w DM)	n.d.	2.6
Glucose (% w/w DM)	n.d.	5.4
Other monosaccharides (% w/w DM)	n.d.	11.4
Other disaccharides (% w/w DM)	n.d.	4.7
Protein (% w/w DM)	0.1 ± 0.0	17.1± 0.88
Mineral (mg/g DM)	24.6	112
Main elements (mg/g DM)		
Sodium	3.2	5.9
Magnesium	1.0	20.1
Potassium	6.3	84.7
Calcium	9.8	0.6
Phosphorus	4.2	0.7
Minor elements (μg/g DM)		
Boron	17	69
Iron	n.d.	83
Nickel	n.d.	13
Copper	n.d.	48
Zinc	5	53
Rubidium	7	33
Strontium	4	n.d.
**pH**	5.4	4.5

^a^ Data according to Díez-Municio et al. [19].

^b^n.d.: not detected.

The main metal ions identified in CWP were those derived from the most abundant mineral salts in it, including calcium, magnesium, sodium, potassium and phosphorus. Besides of these, trace minerals as boron, zinc, rubidium and strontium were also detected [19].

The most abundant cations in the TW were potassium and magnesium, which was expectable due to the high content in potassium of legumes and the addition of magnesium chloride as protein coagulant agent during tofu manufacturing [23]. Likewise, other abundant ions, although in minor amount, as calcium, phosphorus and sodium, as well as trace minerals, including boron, iron, nickel, copper, rubidium and zinc were also detected in the TW (Table 2).

Regarding the total content in soluble proteins, this was very low in the CWP (0.1%, w/w DM), due to the previous process of ultrafiltration membrane at which cheese whey is subjected to concentrate whey proteins. However, TW showed higher protein content, ~17% (w/w DM), which is according with the scarce data previously described in the literature [23].

#### Characterization and quantification of the carbohydrate profile

The characterization and quantitation of carbohydrates present in both by-products is highly important, since these will be the starting substrates of the enzymatic reaction and, hence, will determine the yields obtained for lactosucrose synthesis.

**Fig 1:**
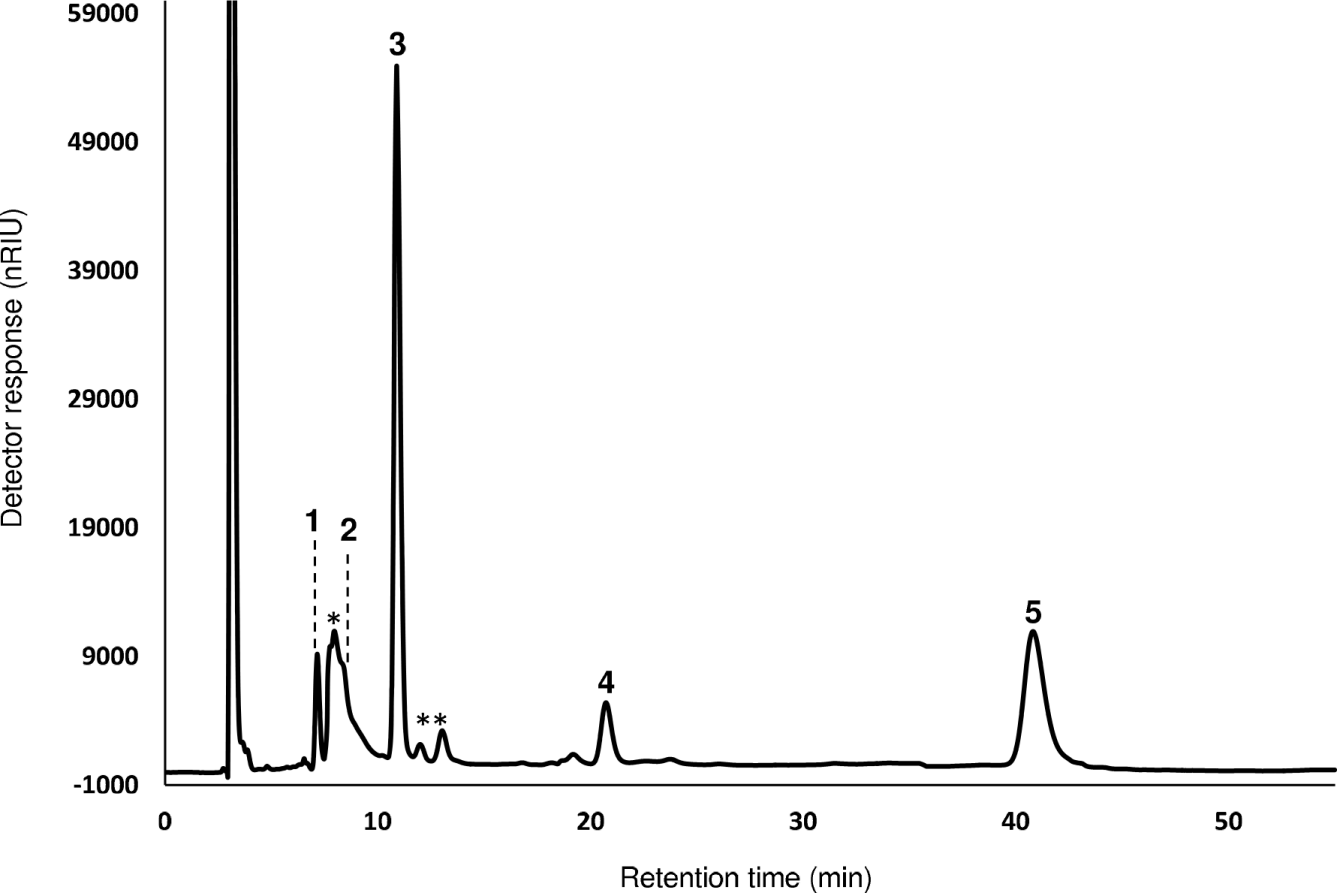
Chromatographic profile of carbohydrates present in tofu whey obtained by LC-RID. 1 = fructose; 2 = glucose; 3 = sucrose; 4 = raffinose; 5 = stachyose; * other monosaccharides; ** = other disaccharides.

As it was expected, chromatographic profile of CWP showed only one peak corresponding to lactose (89.9%, w/w DM) (chromatogram not shown), whilst TW showed a more complex pattern with two major peaks identified as sucrose (peak 3, t_R_ = 10.9 min, 21%, w/w DM) and stachyose, O-α-D-galactopyranosyl-(1–6)O-α-D-galactopyranosyl(1–6)-O-α-D-glucopyranosyl-(1–2)-β-D-fructofuranoside, (peak 5, t_R_ = 40.5 min, 16.3%, w/w DM) (Fig 1), in agreement with the characterization of tofu whey previously carried out [23]. Both carbohydrates should be the main fructosyl donors during enzymatic synthesis of lactosucrose. A less intense peak corresponding to raffinose, (O-α-D-galactopyranosyl(1–6)-O-α-D-glucopyranosyl-(1–2)-β-D-fructofuranoside), the third of the carbohydrates acting as a plausible reaction substrate, was also detected (peak 4, t_R_ = 20.5 min, 3.7%, w/w DM). LC chromatogram also showed two minor peaks identified as fructose (peak 1, t_R_ = 7.1 min, 2.6%, w/w DM) and glucose (peak 2, t_R_ = 7.8 min, 5.4%, w/w DM), as well as several non-identified peaks (indicated by an asterisk) eluting in the mono- and disaccharide area (11.4% and 4.7%, w/w DM, respectively) (Fig 1 and Table 2).

### Enzymatic synthesis of lactosucrose by using model systems

A number of studies have demonstrated the potential of sucrose to act as donor of fructose molecules during the biotechnological synthesis of lactosucrose catalyzed by levansucrases with microbial origin as it was recently reviewed [16]. However, as far as we know, the yield of lactosucrose production using raffinose or stachyose as fructosyl donors, in the presence of lactose as acceptor, has not been studied until now. These oligosaccharides, as galactosylated derivatives of sucrose, might also act as donors of fructose units in reactions catalyzed by levansucrases. In fact, several microbial levansucrases have been reported to use raffinose as both a donor and an acceptor of fructosyl moieties to synthetize mostly polymers of the levan type [28–30], as well as a series of oligosaccharides with a degree of polymerization up to 6 [31]. Nonetheless, to the best of our knowledge, the capacity of raffinose and stachyose to act as fructosyl donors in transfructosylation reactions catalyzed by the levansucrase of *B*. *subtilis* CECT 39 has not been studied up to date.

**Fig 2:**
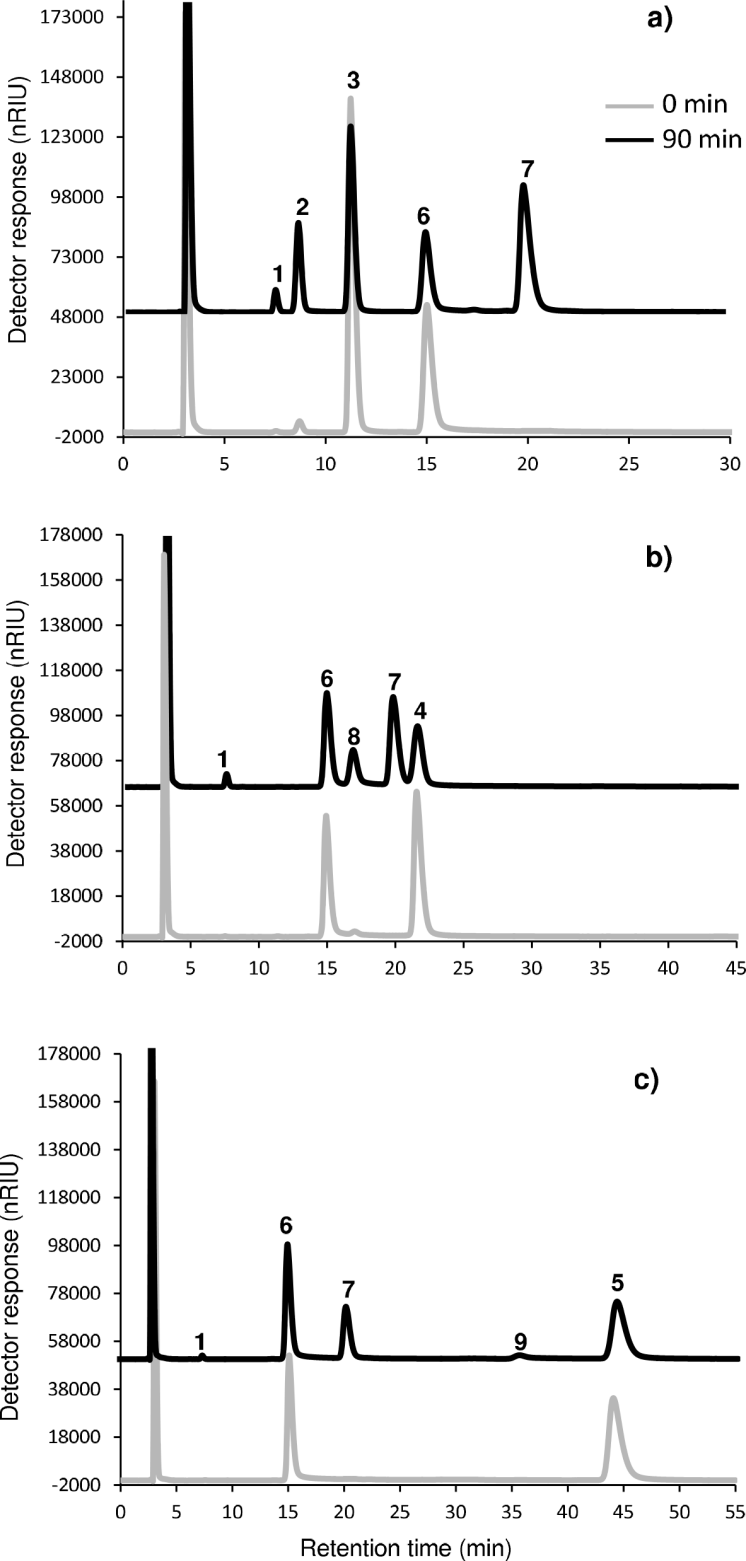
LC-RID profiles of transfructosylation reactions based on: a) sucrose:lactose, b) raffinose:lactose and c) stachyose:lactose mixtures (25%:25%) catalyzed by levansucrase from *B*. *subtilis* CECT 39 (0.5 U mL^-1^) at 37°C, in 50 mM potassium phosphate buffer at pH 6.0, for 0 and 90 min. 1 = fructose; 2 = glucose; 3 = sucrose; 4 = raffinose; 5 = stachyose; 6 = lactose; 7 = lactosucrose; 8 = melibiose; 9 = 6’-galactosyl melibiose.

Fig 2A shows the LC-RID chromatogram obtained for the enzymatic reaction mixture at 0 and 90 min with Suc:Lac mixture at a concentration of 25%:25% (w/v). At the initial time, only two well-resolved peaks, corresponding to sucrose (peak 3, t_R_ = 11.2 min) and lactose (peak 6, t_R_ = 15 min) were observed. However, after 90 min of reaction, three new peaks were clearly detected (peaks 1, 2 and 7), while intensity of peaks corresponding to sucrose and lactose notably decreased, indicating their high capacity as donor and acceptor, respectively. At 90 min of reaction, the less retained peaks corresponded to fructose (peak 1, t_R_ = 7.5 min) and glucose (peak 2, t_R_ = 8.7 min), which were derived from hydrolysis of sucrose. Moreover, glucose was much more abundant than fructose, indicating that fructose was efficiently transferred. The other new peak was identified as lactosucrose (peak 7, t_R_ = 20 min).

Chromatograms obtained after LC-RID analysis of Raf:Lac (Fig 2B) and Sta:Lac (Fig 2C) mixtures at a concentration of 25%:25% (w/v) showed a profile very similar to that of Suc:Lac mixture. Thus, at the initial time, only two major peaks, corresponding to raffinose (peak 4, t_R_ = 21.6 min) or stachyose (peak 5, t_R_ = 44.2 min) and lactose (peak 6, t_R_ = 15 min) were detected, whilst after 90 min of reaction, new peaks appeared as a result of the reaction progress and, concomitantly, peaks of raffinose/stachyose and lactose substantially decreased, indicating their capacity as donors and acceptors, respectively, in transfructosylation reaction. New peaks were identified as fructose (peak 1; t_R_ = 7.5 min; derived from hydrolysis of raffinose/stachyose), melibiose (peak 8; t_R_ = 16.9 min; derived from hydrolysis of raffinose) and an unknown peak (peak 9; t_R_ = 35.4 min; derived from hydrolysis of stachyose), but probably corresponding to the trisaccharide 6’-galactosyl melibiose (α-D-Gal-(1–6)-α-D-Gal-(1–6)-α-D-Glu). This trisaccharide was previously observed [32] after hydrolysis of stachyose by a commercial enzyme preparation (Pectinex Ultra SP-L) with fructosyltransferase activity. The other new peak was identified as lactosucrose (peak 7, t_R_ = 20.7 min), the acceptor reaction product.

**Fig 3:**
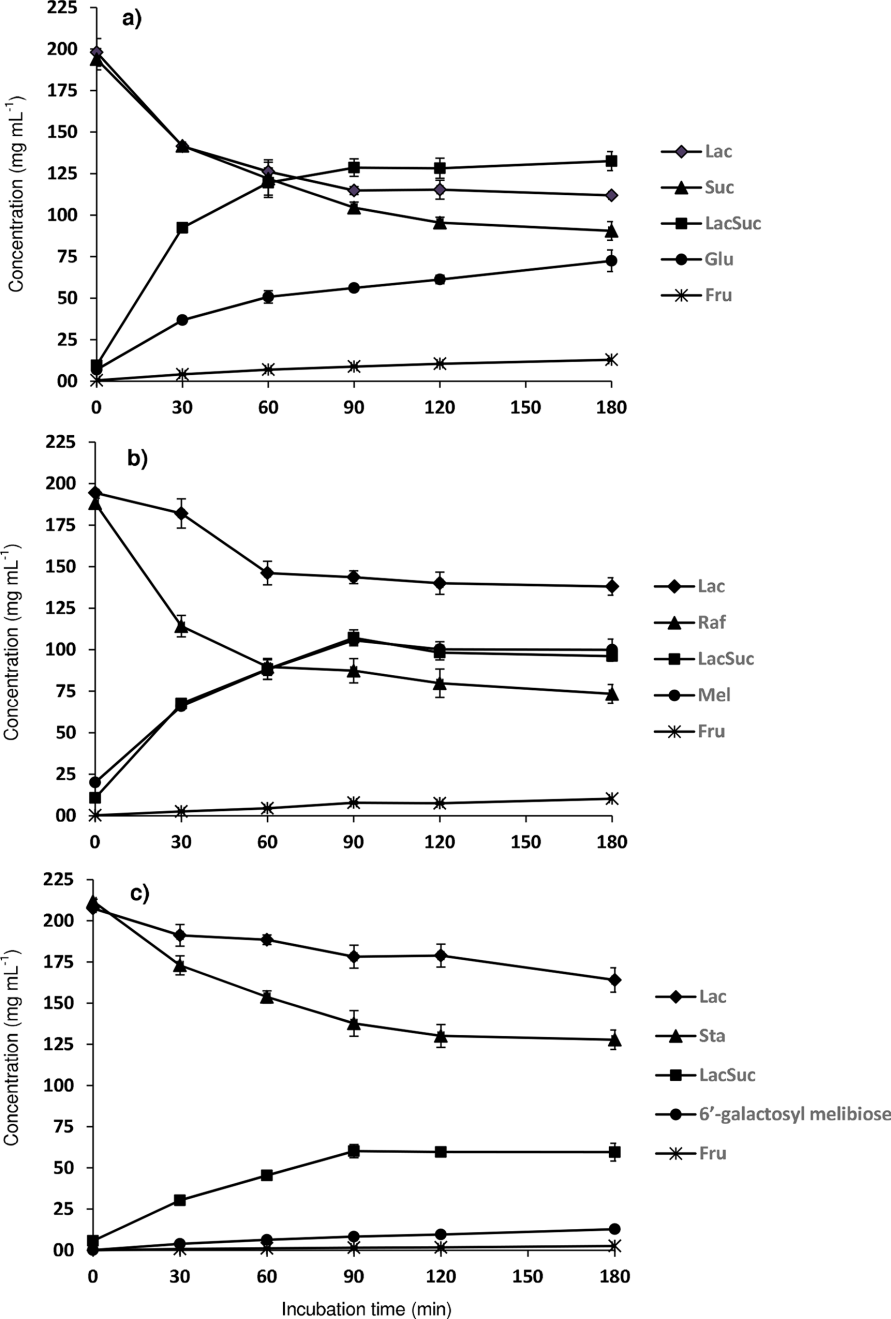
Concentrations of fructose, glucose, sucrose, lactose, raffinose, stachyose, melibiose, 6’-galactosyl melibiose and lactosucrose upon transfructosylation reactions based on: Suc:Lac (a), Raf:Lac (b), and Sta:Lac (c) mixtures (25%:25%) catalyzed by levansucrase from *B*. *subtilis* CECT 39 (0.5 U mL^-1^) at 37°C, in 50 mM potassium phosphate buffer at pH 6.0, for 180 min. Vertical bars represent standard deviations (n = 4).

Fig 3 shows the evolution of the content in sucrose, raffinose, stachyose, lactose, fructose, glucose, melibiose, 6’-galactosyl melibiose and lactosucrose with the reaction time. For all the tested donors (Suc, Raf and Sta), the maximum formation of lactosucrose was achieved after 90 min of transfructosylation reaction and, then, it remained practically constant until the end of the enzymatic reaction. As commented above, this increase and subsequent plateau coincided with the gradual decrease of lactose, as well as sucrose (Fig 3A), raffinose (Fig 3B) and stachyose (Fig 3C) up to 180 min of reaction. Thus, a loss of 46% of sucrose, 54% of raffinose and 35% of stachyose in weigh respect to the initial amount was observed after 90 min of reaction, confirming the different ability of the levansucrase to hydrolyze these carbohydrates to act as donor molecules. In addition, lactose concentration decreased 42%, 26% and 14% when sucrose, raffinose and stachyose were acting as donors, respectively, indicating the capacity of lactose to serve as acceptor for the formation of the main trisaccharide, that is lactosucrose.

Lactosucrose concentration, yield, productivity and specific productivity (determined after 90 min of reaction) were different depending on the donor affinity, being as follows: sucrose (128.6 g L^-1^, 65%, 85.7 g L^-1^ h^-1^ and 171.4 mg U enzyme^−1^ h^−1^) > raffinose (107.1 g L^-1^, 55%, 71.4 g L^-1^ h^-1^ and 142.8 mg U enzyme^−1^ h^−1^) > stachyose (60.2 g L^-1^, 29%, 40.1 g L^-1^ h^-1^ and 80.2 mg U enzyme^−1^ h^−1^). This could be related to that, probably, the ability to donate fructosyl units of raffinose and, specially, stachyose is limited as compared to sucrose due to their higher steric hindrance in the enzyme active site.

### Enzymatic synthesis of lactosucrose by using the industrial by-products as substrates

#### Synthesis from tofu whey (donor) and lactose (acceptor)

Based on data derived from the quantification of the carbohydrate profile of TW (Fig 1 and Table 2), the amount of TW equivalent to 25% of Suc, Raf and Sta was estimated. Nevertheless, due to the insolubility of the resulting reaction mixture, that is TW:Lac (57.4%:25%, w/v), it was necessary to reduce the concentration of TW and/or lactose. After several solubility assays (data not shown), two conditions were selected: (i) TW:Lac (51.6%:25%), 51.6% of TW being equivalent to 9.7% Suc, 1.7% Raf and 7.5% Sta. This mixture was called TW1 from now on; and ii) the mixture named TW2 based on TW:Lac (47.3%:12.5%); 47.3% of TW being equivalent to 9% Suc, 1.6% Raf and 7% Sta.

**Fig 4:**
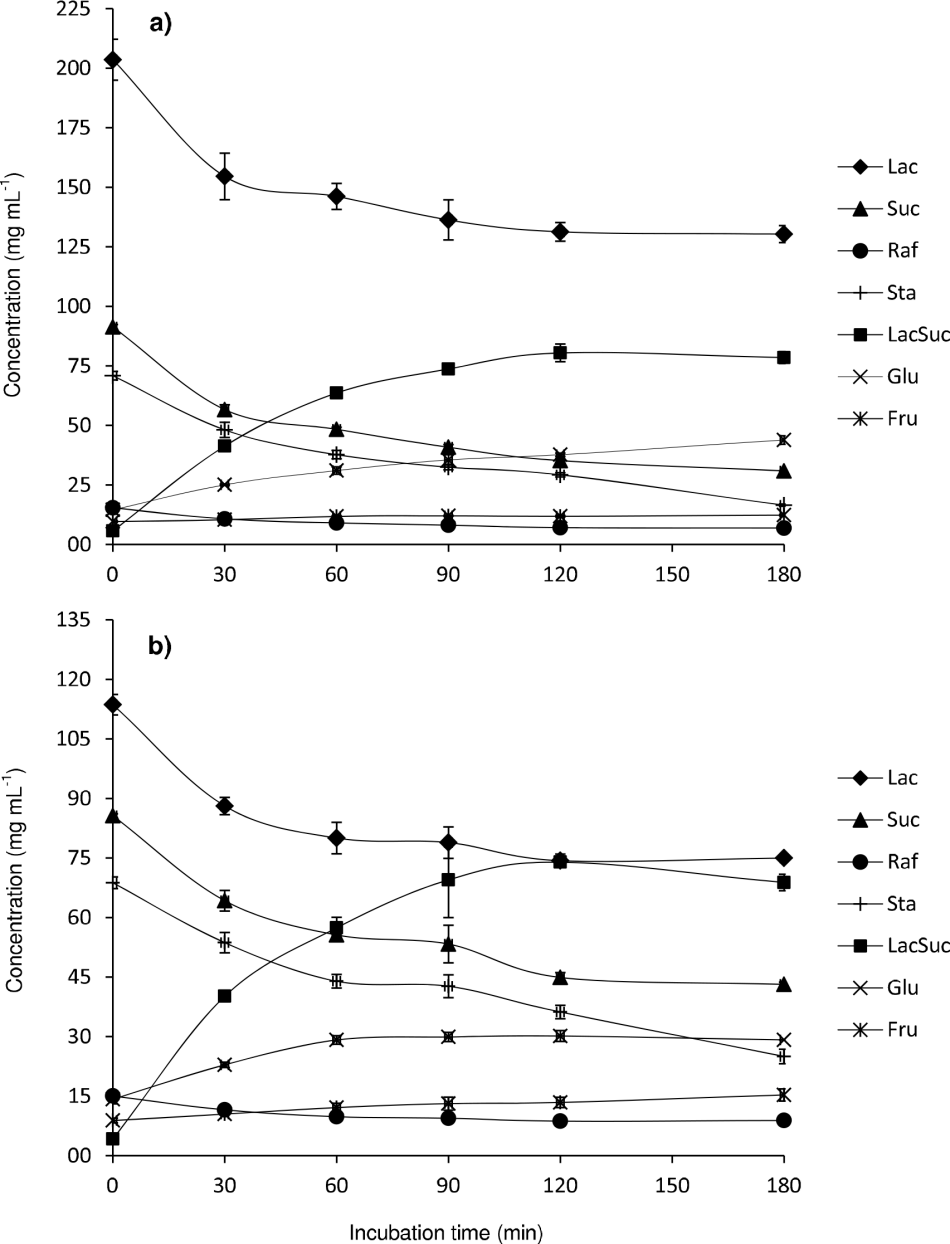
Concentrations of fructose, glucose, sucrose, lactose, raffinose, stachyose and lactosucrose upon transfructosylation reactions based on: TW:Lac (51.6%:25%) (a) and TW:Lac (47.3%:12.5%) (b) mixtures catalyzed by levansucrase from *B*. *subtilis* CECT 39 (0.5 U mL^-1^) at 37°C, in 50 mM potassium phosphate buffer at pH 6.0, for 180 min. Vertical bars represent standard deviations (n = 4).

Fig 4 displays the evolution of the content in sucrose, raffinose, stachyose, lactose, fructose, glucose and lactosucrose with the reaction time. Either for TW1 (Fig 4A) or TW2 (Fig 4B), the maximum formation of lactosucrose was achieved after 120 min of reaction, this increase in lactosucrose concentration was concomitant with the gradual decrease of lactose and sucrose, raffinose and stachyose, indicating the potential use of tofu whey as donor in the enzymatic reaction of lactosucrose formation and the contribution of not only sucrose but also raffinose and stachyose as donors of fructosyl moieties.

The formation of lactosucrose was efficient in both reaction mixtures, TW1 and TW2, and fairly similar values in lactosucrose production, i.e. 80.5 and 74 g L^-1^, were obtained after 120 min of reaction, respectively. However, taking into account the lower initial amount of Lac in TW2 as compared to TW1, the purity of lactosucrose was slightly superior in the former (27.9% in TW2 vs. 25.2% in TW1); whilst the yield of lactosucrose formation, by weight with respect to the initial amount of lactose, was markedly higher with the mixture TW2 (64.9%) than with TW1 (39%). Additionally, the analysis of variance (ANOVA) test indicated that lactosucrose values obtained at 3 hours of enzymatic reaction were not significantly different (P > 0.05) from those determined after 2 hours.

Finally, and in order to obtain a better comparison with the lactosucrose synthesis using model systems, productivity and specific productivity values obtained after 120 min of transfructosylation reaction were as follows: 40.3 g L^-1^ h^-1^ and 80.6 mg U enzyme^−1^ h^−1^ for TW1; 37.0 g L^-1^ h^-1^ and 74 mg U enzyme^−1^ h^−1^ for TW2.

#### Synthesis from tofu whey (donor) and cheese whey permeate (acceptor)

Finally, with the purpose of studying the capacity of the CWP as acceptor in the transfructosylation reaction, enzymatic reactions were performed by using two different mixtures TW:CWP with the same concentration of TW than TW1 and TW2 and with a content in CWP equivalent to an initial lactose concentration similar to TW1 and TW2: (i) TW:CWP (51.6%:27.8%), 27.8% of CWP being equivalent to 25% of lactose. This mixture was called TCW1 (tofu and cheese whey 1); and ii) TW:CWP (47.3%:13.9%). 13.9% of CWP being equivalent to 12.5% of lactose. This mixture was called TCW2 (tofu and cheese whey 2).

**Fig 5:**
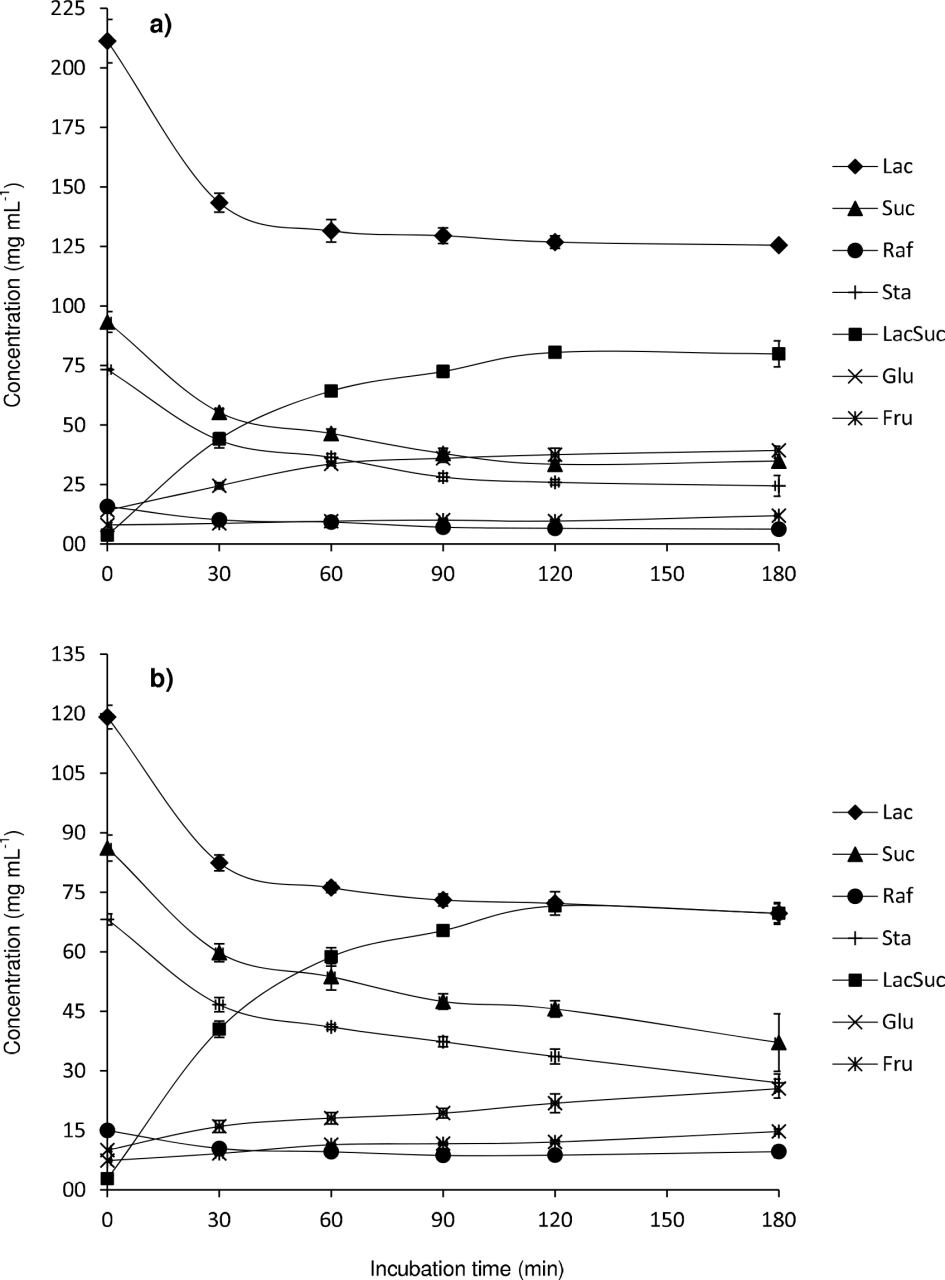
Concentrations of fructose, glucose, sucrose, lactose, raffinose, stachyose and lactosucrose upon transfructosylation reactions based on: TW:CWP (51.6%:27.8%) (a) and TW:CWP (47.3%:13.9%) (b) mixtures catalyzed by levansucrase from *B*. *subtilis* CECT 39 (0.5 U mL^-1^) at 37°C, in 50 mM potassium phosphate buffer at pH 6.0, for 180 min. Vertical bars represent standard deviations (n = 4).

Evolution of the content in sucrose, raffinose, stachyose, lactose, fructose, glucose and lactosucrose with the reaction time is shown in Fig 5. As occurred with TW1 and TW2 samples, the maximum formation of lactosucrose was achieved after 120 min of the reaction with both TCW1 (Fig 5A) and TCW2 (Fig 5B). Such increase coincided with the gradual decrease of lactose, indicating that lactose present in CWP is able to accept molecules of fructose released during the hydrolysis of sucrose, raffinose and stachyose of tofu whey. Moreover, the maximum formation of lactosucrose achieved a plateau from the second hour of reaction, remaining fairly constant until the end of the reaction (Fig 5), which could be an advantage when operating at large-scale. This result was statistically confirmed by an analysis of variance (ANOVA) with lactosucrose values obtained after 2 and 3 hours of reaction not being significantly different (P > 0.05).

The maximum yield of lactosucrose formation (in weight respect to the initial amount of lactose) and production from mixture TCW1 were 38.5% and 80.1 g L^-1^, and 60.8% and 71.5 g L^-1^ for TCW2, which were very similar values to those obtained from TW1 and TW2, respectively. Likewise, lactosucrose purity was also higher for TCW2 (28.2%) than for TCW1 (24.8%). Moreover, productivity and specific productivity values obtained after 120 min of transfructosylation reaction for TCW1 and TCW2 samples resulted to be 40.1 g L^-1^ h^-1^ and 80.1 mg U enzyme^−1^ h^−1^, and 35.8 g L^-1^ h^-1^ and 71.5 mg U enzyme^−1^ h^−1^, respectively.

**Fig 6:**
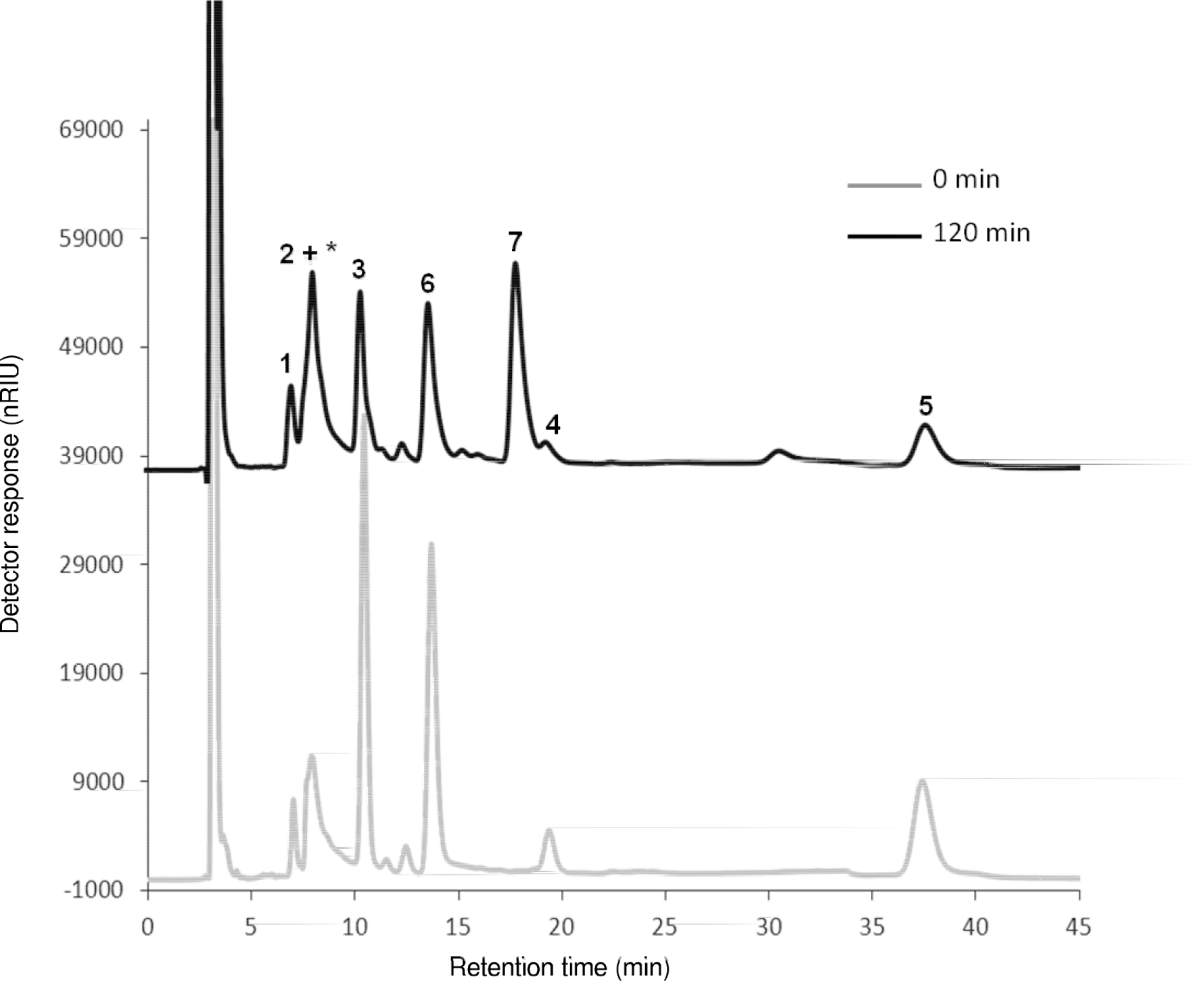
LC-RID profiles of transfructosylation reactions based on: TW:CWP (47.3%:13.9%) (TCW2) mixture catalyzed by levansucrase from *B*. *subtilis* CECT 39 (0.5 U mL^-1^) at 37°C, in 50 mM potassium phosphate buffer at pH 6.0, for 0 and 120 min. 1 = fructose; 2 = glucose; 3 = sucrose; 4 = raffinose; 5 = stachyose; 6 = lactose; 7 = lactosucrose; * other monosaccharides.

LC-RID profiles corresponding to TCW2 at 0 and 120 min clearly illustrated the area decrease of peaks corresponding to sucrose (peak 3), raffinose (peak 4), stachyose (peak 5) and lactose (peak 6) with the increasing lactosucrose concentration (peak 7) (Fig 6).

Results contained in this work are the first evidence, to the best of our knowledge, of the production of lactosucrose by using a combination of by-products (tofu whey and cheese whey permeate), as well as, stachyose:lactose and raffinose:lactose model systems. Nevertheless, to conduct an efficient upgrading of the studied by-products, it is necessary to produce lactosucrose with an acceptable yield as compared to those obtained with standard buffered solutions of lactose and sucrose. In this context, the productivity values obtained by using tofu whey and cheese whey permeate at two different weight ratios as precursor substrates for lactosucrose synthesis (i.e., 35.8 and 40.1 g L^-1^ h^-1^) were: i) much higher than those described for the synthesis of lactosucrose from standard lactose/sucrose solutions and catalyzed by cells from *Paenibacillus polymyxa* IFO 3020 [33], *Bacillus subtilis* KCCM 32835 [34] or by a recombinant enzyme produced by *Bacillus subtilis* NCIMB 11871 [35]; ii) similar to those described by Han et al. [36] by using a recombinant enzyme from *Zymomonas mobilis*; or iii) lower than data reported by Han et al. [37] who used a huge concentration of starting substrates (i.e., 510 g L^-1^ of sucrose and 360 g L^-1^ of lactose) and a higher reaction temperature, 45°C, than that used in our work. Therefore, in terms of productivity and yield values, as well as according to short reaction time and moderate temperature needed to produce lactosucrose, the proposed procedure is a novel bioprocess method to upgrade two important and inexpensive agro-food by-products, such as tofu whey and cheese whey permeate.

On the other hand, despite the use of agro-industrial by-products having a more complex composition as compared to lactose and sucrose (Table 2), the enzymatic synthesis of lactosucrose is highly regio- and stereoselective leading to a non-complex mixture of oligosaccharides (Fig 6). Therefore, the production of lactosucrose from tofu and cheese whey permeate could not represent additional drawbacks for its subsequent isolation and purification as compared to the procedure followed at high scale. Nevertheless, it should bear in mind that minerals and proteins might have a negative effect in the operation and lifespan of the chromatography, membrane and/or (ab)/adsorption equipment.

At industrial level, lactosucrose is produced through a transfructosylation reaction catalyzed by a β-fructofuranosidase from a specific soil bacterium (i.e., *Arthrobacter* sp. K-1) using sucrose and lactose as starting substrates [38]. In addition to the use of an invertase-deficient yeast for removing monosaccharides (mainly glucose), the reaction mixture is then purified by decoloration, carbonation, filtration, desalination, ultrafiltration and concentration to produce syrups containing up to 55% of lactosucrose. This same purification protocol could also potentially be applied to the resulting mixture obtained from the production of lactosucrose using cheese and tofu whey described in the current work. Additionally, as lactosucrose is the only trisaccharide present in the reaction mixture (apart from minor levels of raffinose and trace amounts of 6’-galactosyl melibiose), large-scale size-exclusion chromatography could be an interesting alternative for the isolation of lactosucrose with a high-purity.

### Structural confirmation of synthetized lactosucrose by GC-MS

**Fig 7:**
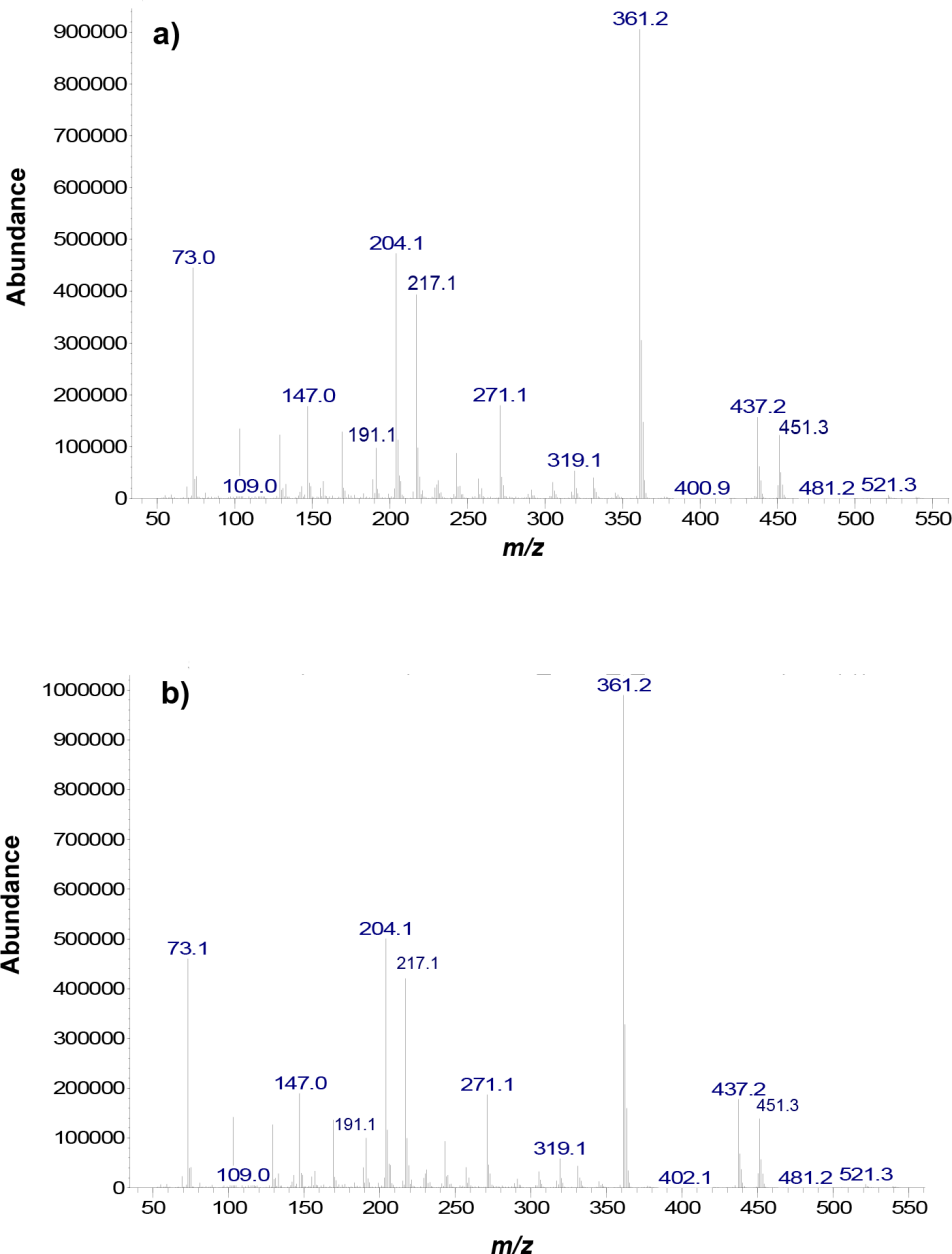
Mass spectra obtained by gas chromatography coupled to mass spectrometry (GC-MS) analysis using the corresponding trimethylsilyl oximes (TMSO) of the lactosucrose synthetized from TCW2 mixture at 120 min (a) and the commercial lactosucrose standard (b).

The identification of synthetized lactosucrose was made by comparison with the respective GC retention index of the commercial standard. Furthermore, structural confirmation of lactosucrose was achieved by comparison of the mass spectrum corresponding to synthetized lactosucrose (from TCW2 mixture at 120 min) with the respective MS of the commercial standard (Fig 7). The most abundant ion was that at *m/z* 361 which is characteristic of a glycosylated sugar ring [39, 40] and it has been previously described in lactosucrose [41]. This stable ion is originated from the loss of a trimethylsilanol group (TMSOH; *m/z* = 90) from the pyranose moiety at *m/z* = 451, also observed in lactosucrose spectrum. Fragmentation behavior of lactosucrose unit also resulted in i) ion at *m/z* 437 which is specific from ketohexoses in both pyranose and furanose forms, free and mono-substituted [40]; ii) ion at *m/z* 271 derived from the loss of a TMSOH group of the ion at *m/z* = 361 [41]; iii) ion at *m/z* 204 (typical of pyranose rings), which is originated from two neighboring units of TMSOCH, except from C6 and C1; and iv) ion at *m/z* 217 (typical of furanose rings), containing two units of TMSOCH from C6 and C5 completed by the atom of C4 [40].

## Conclusions

This work describes a simple and fast process developed at a moderate temperature, 37°C, for the efficient synthesis of the potential prebiotic oligosaccharide lactosucrose from two abundant and inexpensive industrial by-products; tofu whey and cheese whey permeate, which are rich in sucrose, raffinose and stachyose (fructosyl donors) and lactose (acceptor), respectively, by a transfructosylation reaction catalyzed by the levansucrase from *B*. *subtilis* CECT 39. Our results demonstrated the ability of other substrates apart from sucrose, i.e. raffinose and stachyose, to efficiently participate in the synthesis of lactosucrose. Both by-products were suitable substrates for the high-yield synthesis of lactosucrose with a maximum production of 80.1 g•L^-1^ after a short reaction time (120 min).

To conclude, these findings could provide a new strategy to valorize agro-industrial by-products as cheese whey permeate and tofu whey by means of their use as renewable substrates for conversion into lactosucrose, a bioactive oligosaccharide with a high value-added.
